# Supplementary material for: MetaRibo-Seq measures translation in microbiomes
Source: Nat Commun. 2020 Jun 29;11:3268. doi: 10.1038/s41467-020-17081-z (PMC7324362; doi:10.1038/s41467-020-17081-z)
Supplement: Supplementary file 10 — Supplementary Data 7 [file 41467_2020_17081_MOESM10_ESM.zip › File2/Confidence_High_Taxonomy/157190_out.krona.html]

Javascript must be enabled to view this page.

members
magnitude
magnitudeUnassigned
count
unassigned
taxon
rank

157190\_out

2

2
2759
superkingdom

kingdom
2
4751

2
1913637
phylum

subphylum
214504
2

class
2
214506

2
36750
order

family
36751
2

2
1129544
genus


SRS013687\_contig\_number\_14931SRS1041145\_contig\_number\_28442
species
2
588596
